# Global and local process on the Rey Complex Figure Test in older adults

**DOI:** 10.1002/alz.087211

**Published:** 2025-01-03

**Authors:** Eun Hyun Seo, Hyung‐Jun Yoon, Seung Gon Kim, Kyu Yeong Choi, Jung‐Min Ha, Hoowon Kim, Kun Ho Lee

**Affiliations:** ^1^ Gwangju Alzheimer’s & Related Dementias (GARD) Cohort Research Center, Gwangju Korea, Republic of (South); ^2^ College of Medicine, Chosun University, Gwangju Korea, Republic of (South); ^3^ Department of Neuropsychiatry, Chosun University Hospital, Gwangju Korea, Republic of (South); ^4^ Gwangju Alzheimer’s and Related Dementia (GARD) Cohort Research Center, Chosun University, Gwangju Korea, Republic of (South); ^5^ Department of Nuclear Medicine, School of Medicine, Chosun University/Chosun University Hospital, Gwangju Korea, Republic of (South); ^6^ Department of Neurology, School of Medicine, Chosun University/Chosun University Hospital, Gwangju Korea, Republic of (South)

## Abstract

**Background:**

Recent studies have shown that the Rey Complex Figure Test (RCFT) is useful for early detection of Alzheimer’s disease (AD). While RCFT is known as a test for visuoconstruction and memory, it demands executive components during copying and recalling of the complex figure. This study aims to explore which features of executive function are involved in RCFT performance and which of them are clinically significant indicators in older adults.

**Method:**

We included 4540 participants (2384 cognitively normal, 272 subjective cognitive decline, 1662 mild cognitive impairment, 222 dementia) from the GARD Cohort in Korea. Based on the qualitative analysis by Cherrier et al (1999), we further divided the RCFT score into six groups (gestalt/ detail/ left/ right/ upper/ lower) to explore specific executive component. The RCFT subscores were compared between groups using ANCOVAs. Partial eta squared *(η^2^_p_*) was calculated for effect size. Linear regression analysis with stepwise selection was performed to examine associations between the RCFT subscores and other neuropychological scores.

**Result:**

The six subscores from copy, immediate recall, and delayed recall trials were all significantly different among CN, SCD, MCI, and dementia groups. Based on *η^2^_p,_* ‘detail’ score on copy trial (*η^2^_p,_* = 0.228) ‘gestalt’ scores on memory trials (*η^2^_p,_* = 0.185 for delayed; *η^2^_p,_* = 0.213 for delayed) showed largest effects. In terms of relations to cognitive domain, for copy trial, both ‘gestalt’ and ‘detail’ scores were associated with tests of psychomotor (*ß* = 0.074∼0.193, all *p* <0.0001), delayed verbal memory (*ß* = 0.067∼0.077, all *p* <0.0001), and executive functions (*ß* = ‐0.176∼‐0.185, all *p* <0.0001). For memory trials, ‘gestalt’ scores were mainly associated with tests of executive functions (*ß* = ‐0.069∼‐0.100, all *p* <0.0001), whereas, ‘detail’ scores were mainly associated with tests of psychomotor and attention (*ß* = ‐0.073∼‐0.144, all *p* <0.0001).

**Conclusion:**

Our results suggest that the RCFT performance involves cognitive process regarding strategic planning and organization of a fractional figure into whole object. In particular, local process on copy trial, measured by ‘detail’ score and global process on memory trials, measured by ‘gestalt’ score may be a useful indicator of early cognitive impairment. It may be necessary to collect procedural features during RCFT performance to capture perceptual organisation strategies.

